# Intravascular lithotripsy for the treatment of calcified coronary lesions in individuals of advanced age: a post-hoc analysis of the multicentre, prospective BENELUX-IVL study

**DOI:** 10.1016/j.eclinm.2025.103342

**Published:** 2025-07-04

**Authors:** Federico Oliveri, Pablo Valentín García, Martijn J.H. van Oort, Akshay Phagu, Ibtihal Al Amri, Brian O. Bingen, Valeria Paradies, Gianluca Mincione, Bimmer E. Claessen, Aukelien C. Dimitriu-Leen, Joelle Kefer, Hany Girgis, Tessel Vossenberg, Alessandro Mandurino-Mirizzi, Frank Van der Kley, J. Wouter Jukema, José M. Montero-Cabezas

**Affiliations:** a Department of Cardiology, Leiden University Medical Center, Leiden, the Netherlands; b Department of Cardiology, Complejo Hospitalario Universitario de Albacete, Spain; c Department of Cardiology, Maasstad Ziekenhuis, Rotterdam, the Netherlands; d Department of Cardiovascular Medicine, Humanitas Research Hospital-IRCCS, Rozzano, Italy; e Department of Cardiology, Amsterdam University Medical Center, the Netherlands; f Department of Cardiology, Radboud University Medical Center, the Netherlands; g Department of Cardiology, Saint-Luc Bruxelles, Belgium; h Department of Cardiology, Jeroen Bosch Ziekenhuis, Den-Bosch, the Netherlands; i Department of Cardiology, Frisius Medisch Centrum, the Netherlands; j Department of Experimental Medicine (DiMeS), University of Salento, Lecce, Italy; k Netherlands Heart Institute, Utrecht, the Netherlands

**Keywords:** Intracoronary lithotripsy, PCI and advanced age, Calcium and advanced age, IVL in elderly

## Abstract

**Background:**

Patients of advanced age are frequently underrepresented or excluded from major clinical trials, leading to limited evidence in this population and a reliance on data extrapolated from younger populations. The present study aims to compare and evaluate the safety and efficacy of intravascular lithotripsy (IVL) for the treatment of calcified coronary lesions in individuals of advanced age and in younger populations.

**Methods:**

In this post-hoc analysis, from the ongoing prospective, multicenter BENELUX-IVL registry involving nine hospitals across four countries in the European Union, patients undergoing percutaneous coronary intervention (PCI) with intravascular lithotripsy (IVL) were retrospectively stratified into two groups: advanced age (≥80 years) and younger (<80 years). The primary technical endpoint was technical success, defined as successful IVL catheter crossing of the target lesion with residual stenosis <30%. The primary safety endpoint was in-hospital major adverse cardiac events (MACE). This study is registered with ClinicalTrials.gov, number NCT06577038.

**Findings:**

Between January 2019 and April 2025, 583 patients underwent PCI with IVL; of these, 147 (25.2%) patients were aged ≥80 years (advanced age cohort), and the remaining 436 (74.8%) were aged <80 years (younger cohort). Compared to younger patients, the advanced age cohort had higher SYNTAX score (23 [IQR 15–32] vs. 18 [IQR 11–28], p = 0.01) and more comorbidities. Intraprocedural complications (severe dissection, abrupt vessel closure, and perforation) were low in both groups, with no significant differences between them. Technical success was comparable between the advanced age and younger populations (87.8% vs. 90.6%, RR: 0.97 (0.91–1.04), p = 0.32). In-hospital MACE rates were similar between the advanced age and younger cohorts (1.4% vs. 2.1%, RR: 0.64 (0.19–2.18), p = 0.59), and these results remained consistent at 1-year follow-up (6.1% vs. 8.0%, RR: 0.76 (0.38–1.55) p = 0.51).

**Interpretation:**

In patients of advanced age, IVL for calcified coronary lesions demonstrates a favorable safety and efficacy profile, with high technical success and low rates of device-related adverse events. Nonetheless, larger studies with extended follow-up are warranted to confirm these findings and establish long-term outcomes.

**Funding:**

This work was funded through a research grant from 10.13039/100031956Shockwave Medical.

**Research in context**

**Evidence before this study**

In December 2024, we published the 1-year follow-up results of our BENELUX-IVL registry. This led us to question whether outcomes in patients of advanced age were comparable to those in the overall population. Recognising that patients of advanced age are frequently underrepresented or excluded from clinical trials, we conducted a literature search in February 2025 across PubMed and Embase, using the keywords “Intracoronary lithotripsy”, “PCI and advanced age”, “calcium and advanced age”, and “IVL in elderly”. From database inception to February 2025, this search yielded no studies specifically focused on intravascular lithotripsy in patients aged ≥80 years. These findings underscore the paucity of evidence regarding IVL in this high-risk, under-studied population.

**Added value of this study**

This study is based on the largest multicentre registry analysis focused on comparing IVL outcomes between patients aged ≥80 years and those <80 years. By including detailed clinical, procedural, and intracoronary imaging data, we provide important insights into the efficacy and safety of IVL in patients of advanced age. Despite higher comorbidity burdens and more complex coronary anatomy (as reflected by higher SYNTAX scores), which place patients of advanced age at higher procedural risk, intraprocedural complications (including severe dissection, abrupt vessel closure, and perforation) were low in both groups, with no significant differences between them. Moreover, technical success, as well as short-term and long-term MACE and cardiac death rates, were comparable across age groups.

**Implications of all the available evidence**

The combined evidence suggests that IVL is a feasible and effective strategy for treating severely calcified coronary lesions in patients of advanced age, with outcomes comparable to those in the younger population. These findings support the broader use of IVL in routine clinical practice across age groups, including carefully patients of advanced age, who should not be excluded solely because of their age. To confirm these findings and establish long-term outcomes, larger studies with extended follow-up are needed.

## Introduction

With increasing life expectancy, the number of patients of advanced age who require percutaneous coronary intervention (PCI) is rising.^1^ It is estimated that one-third of all PCIs are performed in patients over 75 years of age.^2^ However, performing PCI in this special cohort presents two major challenges. First, this population often has multiple comorbidities, placing them at a higher risk of periprocedural complications.^3^, ^4^, ^5^ Second, the complexity of coronary artery disease (CAD) is typically greater in patients of advanced age. Despite their growing epidemiological significance, patients of advanced age—particularly those over 80 years-old—are frequently underrepresented or excluded from clinical trials, leading to limited evidence and a reliance on data extrapolated from younger populations.

One of the most significant additional procedural challenges in the patients of advanced age undergoing PCI is coronary artery calcification (CAC), which is strongly age-related and an independent predictor of mortality and MACE.^6^, ^7^, ^8^, ^9^ CAC affects more than 90% of men and 70% of women over 70 years of age, often necessitating atherectomy or other specialized lesion preparation devices to achieve optimal procedural outcomes.^6^, ^7^, ^8^, ^9^ In balloon-crossable calcified lesions, intravascular lithotripsy (IVL) has emerged as a safe and effective strategy, offering a low risk of procedural complications.^10^ IVL delivers ultrasound energy through a balloon-based system, inducing superficial and deep calcium fractures, thereby improving vessel compliance and facilitating optimal stent expansion and luminal gain.^11^^,^^12^

The present study aims to compare the safety and efficacy of IVL for the treatment of calcified coronary lesions in advanced age (≥80 years old) and younger age (<80 years old), using data from a real-world registry.

## Methods

### Population and data collection

The BENELUX-IVL registry is an international, multicentre, prospective study (NCT06577038) involving all-comer patients aged ≥18 years who underwent IVL during PCI at nine high-volume PCI centers, starting in January 2019. From the overall registry, patients were retrospectively categoris ed into two groups^1^: patients 80 years or older; and^2^ patients younger than 80 years-old. The considered age was at the time of the procedure. For all the IVL procedures, the Shockwave Intravascular Lithotripsy Coronary System (Shockwave Medical, Santa Clara, California) was used. Procedural technical decisions—including timing, balloon size, number of pulses, maximum pressure, as well as the use of high-pressure pre- and post-dilation, other debulking devices, and intracoronary imaging—were made at the discretion of the operating physician and systematically recorded in procedural documentation. We collected demographic, procedural, clinical, and follow-up data from the hospital's electronic health records. Patients who could not provide informed consent were excluded from the study. Angiographic and imaging data were analys ed in a centralized core laboratory. Patients provided consent for inclusion in the BENELUX-IVL registry and for the use of their anonymized clinical data in research. The study was exempted by the MedicalResearch Ethics Committee Leiden Den Haag Delft (reference number: N22.199/HL/hl), and the retrospective analysis of clinically collected data was approved by the local ethical committees at each participating centre. The registry protocol is available online at ClinicalTrials.gov (NCT06577038).

### Definitions and imaging analysis

In accordance with the most recent European Association of Percutaneous Cardiovascular Interventions (EAPCI) consensus, coronary calcification severity was defined using intravascular ultrasound (IVUS) score (360-degree calcification, calcium extending over 270-degrees and measuring ≥5 mm in length, detection of a calcified nodule, or a vessel diameter less than 3.5 mm) or optical coherence tomography (OCT) score (2 points for a maximum angle greater than 180°, 1 point for a maximum thickness greater than 0.5 mm, and 1 point for a length greater than 5 mm).^13^, ^14^, ^15^ If intravascular imaging was not available, the severity of calcification was assessed angiographically. Radiopaque densities visible without cardiac motion before contrast injection, affecting both sides of the coronary artery wall, were considered a hallmark of severe calcification.^13^ Quantitative coronary analysis (QCA) and intracoronary imaging were retrospectively assessed offline for vessel and stent characteristics. QCA was carried out pre- and post-IVL, using Medis Suite QCA (2D/3D) software (Medis Suite 4.0.24.4; Medis Medical Imaging System BV, Leiden, The Netherlands) by blinded operators. Measurements included minimum lumen diameter (MLD), minimum lumen area (MLA), reference vessel diameter (RVD), percent area stenosis and acute gain. Analysis of IVUS and OCT were performed using QCU-CMS 4.69 (Leiden University Medical Center, Leiden, The Netherlands). Intracoronary imaging parameters were evaluated based on the EAPCI consensus on the clinical use of intracoronary imaging.^16^ Intracoronary imaging measurements included the reference vessel diameter (RVD), area stenosis, pre-PCI minimum lumen area (MLA), max Ca2+ angle, post-PCI minimum stent area (MSA). The asymmetry index (AI) was calculated by subtracting the minimum lesion diameter from the maximum lesion diameter at the MLA or MSA, and then dividing this difference by the maximum lesion diameter. Stent expansion (SE) at the MSA was assessed by dividing the MSA by the reference vessel area.

### Study endpoints

The primary technical endpoint was technical success, defined as the successful delivery of the IVL catheter across the target lesion and delivery of pulses and residual target lesion less than 30% (assessed by QCA) without angiographic complications. The primary safety endpoint was in-hospital MACE, including cardiac death, non-fatal myocardial infarction (MI), or target vessel revascularization (TVR). Secondary technical endpoints included procedural success, defined as technical success, a final Thrombolysis In Myocardial Infarction grade 3 flow, and the absence of in-hospital major adverse cardiac events (MACE). Secondary safety endpoints included MACE, cardiac death, and TVR at a 12-month follow-up.

### Statistical analysis

The primary objective of this analysis was to compare clinical characteristics, procedural details, and outcomes between patients aged ≥80 years and those <80 years. To achieve this, baseline characteristics, procedural variables, and intracoronary imaging findings were compared between groups using appropriate statistical tests. Additionally, a subgroup analysis was performed comparing patients aged ≥80 years, ≤65 years, and those between 65 and 80 years. Continuous variables are reported as either mean ± standard deviation or median with interquartile range (25th–75th percentile), based on their distribution. Normality was assessed by drawing Q–Q plots and checked by the Shapiro–Wilk statistic. Paired continuous variables were evaluated utilizing the paired t-test for normally distributed data and the Wilcoxon signed-rank test for non-normally distributed data. Continuous variables were compared between two groups using the unpaired t-test for normally distributed data and the Mann–Whitney U test for non-normally distributed data. For the subanalysis comparing three age groups (≥80 years, ≤65 years, and 65–80 years), one-way ANOVA was used for normally distributed continuous variables, and the Kruskal–Wallis test was used for non-normal distributions. Categorical variables were expressed as frequencies and percentages and analyzed using the χ2 or Fisher exact test, when appropriate. Key outcomes compared included technical success, procedural success, intraprocedural complications, and in-hospital major adverse cardiac events (MACE). We calculated relative risks (RR) with 95% confidence intervals (CI) for binary outcomes and provided p-values to assess statistical significance. Kaplan–Meier analysis estimated cumulative MACE at 12-month follow-ups. Hazard ratio (HR) was therefore also obtained. Kaplan–Meier were created to visually present and evaluate the results. No upfront power analysis was performed. All tests were two-sided, with p < 0.05 indicating statistical significance. Statistical analyses were conducted using SPSS for Windows, version 25.0 (IBM, Armonk, New York) and R 12.0.

### Role of the funding source

The funder of the study had no role in the study design, data collection, data analysis, or manuscript writing and writing.

## Results

### Baseline characteristics

Between January 2019 and April 2025, a total of 583 patients underwent PCI with IVL; of these, 147 patients were aged ≥80 years, and the remaining 436 were aged <80 years. The baseline characteristics of patients treated with IVL are detailed in Table 1. The proportion of females was similar between the groups (29.3% vs. 25.0%, p = 0.31). Hypertension (78.9% vs. 68.3%, p = 0.02), dyslipidemia (46.3% vs. 56.9%, p = 0.03) and smoking history (31.3% vs 45.0%. <0.01) were higher in the ≥80 years-old group compared to the younger population. Patients in the ≥80 years-old group had a lower estimated glomerular filtration rate compared to those in the <80 years-old group (61 [46–74] vs. 74 [56–89] ml/min, p < 0.01), with a higher prevalence of chronic kidney disease, (44.2% vs. 25.5%, p < 0.01). Coronary artery disease complexity (SYNTAX score) was significantly higher in the ≥80 years-old group (23 [15–32] vs. 18 [11–28], p = 0.01). There were no significant differences in angina severity (p = 0.07). Clinical presentations were heterogeneous, with patients of advanced age more often presenting with NSTEMI.

**Table 1:** Baseline characteristics.

	≥80 years (n = 147)	<80 years (n = 436)	p-value^b^
**Age**, yrs	83 [81–85]	71 [65–75]	**<0.01**
**Female**	43 (29.3)	109 (25.0)	0.31
**BMI**	25.6 [23.7–28.0]	26.8 [24.0–29.6]	0.72
**Hypertension**	116 (78.9)	298 (68.3)	**0.02**
**Dyslipidemia**	68 (46.3)	248 (56.9)	**0.03**
**Smoking history**	46 (31.3)	196 (45.0)	**<0.01**
**Diabetes mellitus**	43 (29.3)	150 (34.4)	0.29
**LV-EF** (%)	50 [39–55]	55 [44–55]	0.06
**Syntax score**	23 [15–32]	18 [11–28]	**0.01**
**Chronic kidney disease** (eGFR <60 ml/min/1.73 m^2^)	65 (44.2)	111 (25.4)	**<0.01**
**GFR** (ml/min)	61 [46–74]	74 [56–89]	**<0.01**
**Previous PCI**	56 (38.1)	209 (47.9)	**0.03**
**Previous CABG**	19 (12.9)	86 (19.7)	0.06
**Previous MI**	41 (27.9)	171 (39.2)	**0.01**
**Previous Stroke/TIA**	28 (19.0)	47 (10.8)	**0.01**
**Clinical presentation**			**0.02**
Stable angina	60 (40.8)	221 (50.7)	
Unstable angina	19 (12.9)	44 (10.1)	
NSTEMI	49 (33.3)	92 (21.1)	
STEMI	7 (4.8)	36 (82.6)	
Other	12 (8.2)	43 (9.9)	
**Angina pectoris**^a^			0.07
No angina	22 (15.0)	52 (11.9)	
Class I	5 (3.4)	16 (3.7)	
Class II	38 (25.9)	163 (37.4)	
Class III	40 (27.2)	100 (22.9)	
Class IV	22 (15.0)	38 (8.7)	
Unknown	19 (12.9)	64 (14.7)	
**Anti-ischemic medication**			
Beta-Blockers	86 (58.5)	264 (60.6)	0.66
Nitrates	42 (28.6)	124 (28.4)	0.97

Values are mean ± SD, median (IQR) or n (%).

eGFR was estimated using the MDRD [Modification of Diet in Renal Disease] formula. BMI, body mass index; CABG, coronary artery bypass grafting; DOAC, direct oral anticoagulants; eGFR, estimated glomerular filtration rate); LV-EF, left ventricle ejection fraction; MI, myocardial infarction; NSTEMI, non ST-elevation myocardial infarction; PCI, percutaneous coronary intervention; STEMI, ST-elevation myocardial infarction; TIA, transient ischemic attack.

Bold values indicate statistical significant.

a According to the Canadian Cardiovascular Society grading of angina pectoris.

b p-values were calculated to compare outcomes between patients aged ≥80 years and those aged <80 years.

### Procedural characteristics

Procedural characteristics of patients treated with IVL for both groups are summarized in Table 2. Procedural time was similar between patients of advanced age and younger population (80 [58–111] min vs. 81 [60–110] min, p = 0.92). Radial access was the preferred approach in both groups (≥80 years-old: 72.8% vs. <80 years: 78.2%, p = 0.31). Regarding target vessels, there was a higher proportion of left main (LM) treatment in the ≥80 years-old group (15.7% vs. 9.4%, p = 0.03). Intraprocedural complications such as severe dissection, abrupt vessel closure, and perforation were low in both groups, with no significant differences observed between both groups. Rate of rotational atherectomy before IVL was similar between both groups (≥80 years-old: 11.6% vs. <80 years-old: 13.3%, p = 0.62). IVL crossing success was high in both groups (95.2% in ≥80 years-old group and 95.6% in <80 years-old group, p = 0.96). Pre-IVL and post-IVL high-pressure balloon dilations were performed in nearly all cases, with no significance between patients of advanced age and younger population. The number of IVL pulses delivered, and IVL balloon diameter did not differ between groups.

**Table 2:** Procedural characteristics.

	≥80 years (n = 147)	<80 years (n = 436)	p-value^a^
**Procedural time** (min)	80 [58–111]	81 [60–110]	0.92
**Contrast volume** (ml)	180 [120–238]	165 [135–230]	0.72
**Inotropes**	8 (5.4)	11 (2.5)	0.08
**Vasopressor**	5 (3.4)	10 (2.3)	0.45
**Need for mechanical support**	5 (3.4)	11 (2.5)	0.56
IABP	0 (0)	2 (0.5)	
Impella	5 (3.4)	6 (1.4)	
VA-ECMO	0 (0)	4 (0.9)	
**Access**			
Radial	107 (72.8)	341 (78.2)	0.31
Femoral	40 (27.2)	105 (81.9)	0.35
**Target lesion**	153	459	
Left main	24 (15.6)	43 (9.4)	**0.03**
Left anterior descending artery	64 (41.8)	163 (3.6)	0.37
Circumflex	22 (14.4)	78 (17.0)	0.37
Right coronary artery	41 (26.8)	171 (37.3)	**0.02**
Venous graft	2 (1.3)	4 (8.7)	0.64
**Bifurcation**	41 (27.8)	94 (21.6)	0.10
**CTO**	4 (2.7)	41 (9.4)	**0.01**
**Ostial lesions**	43 (29.2)	105 (24.1)	0.19
**In-stent**	28 (19.0)	157 (36.0)	**<0.01**
**Rotational atherectomy** (before IVL)	17 (11.6)	58 (13.3)	0.62
**Cutting Balloon** (before IVL)	0 (0)	5 (1.1)	0.19
**Pre-IVL high-pressure dilatation**	140/153 (91.5)	422/459 (91.9)	0.98
**Pre-IVL largest balloon** (mm)	3.0 [2.5–3.5]	3.0 [2.5–3.5]	0.31
**Pre-IVL maximum pressure dilatation** (atm)	18 [16–20]	20 [16–22]	0.46
**IVL crossing success**	140 (95.2)	417 (95.6)	0.96
**IVL pulses delivered**			0.54
<80	78 (53.1)	193 (44.2)	
80	70 (47.6)	215 (49.3)	
>80	5 (3.4)	28 (6.4)	
**Maximum diameter IVL balloon** (mm)	3.5 [3.0–4.0]	3.5 [3.0–4.0]	0.58
**Post-IVL high-pressure dilatation**	140/153 (91.5)	402/436 (92.2)	0.92
**Post-IVL largest balloon** (mm)	3.5 [3.0–4.0]	3.5 [3.5–4.0]	0.67
**Post-IVL maximum pressure dilatation** (atm)	18 [16–20]	20 [16–22]	0.25
**Drug eluting balloon**	9 (6.1)	37 (8.5)	0.38
**Total stent length** (mm)	34 [24–49]	40 [26–60]	**0.01**
**Stent maximum diameter** (mm)	3.5 [3.0–4.0]	3.5 [3.5–4.0]	0.67
**Intraprocedural complications**	10 (6.8)	25 (5.7)	0.62
Severe dissections (D−E−F)	3 (2.0)	8 (1.8)	
Abrupt vessel closure	2 (1.4)	3 (0.7)	
Perforation	2 (1.4)	6 (1.4)	
Need for cover stent	1 (0.7)	5 (1.1)	
Tamponade	0 (0)	1 (0.2)	
Hemodynamic instability (intervention)	4 (2.7)	5 (1.1)	
**Complication IVL-related**	4 (2.7)	3 (0.7)	0.07

Values are mean ± SD or median (IQR).

CTO, chronic total occlusion; IABP, intra-aortic balloon pump; IVL, intravascular lithotripsy; VA-ECMO, veno-arterial extracorporeal membrane oxygenation.

Bold values indicate statistical significant.

a p-values were calculated to compare outcomes between patients aged ≥80 years and those aged <80 years.

### Imaging characteristics

The intracoronary imaging characteristics for patients treated with IVL are detailed in Table 3. Intracoronary imaging was used in approximately half of the patients, and did not differ between groups (≥80 years-old: 47.6% vs. <80 years-old: 54.1%, p = 0.22). However, differences were observed regarding the imaging device used, being IVUS more commonly used in the ≥80 years-old group (98.6% vs. 89.4%, p = 0.01). Post-PCI, there were no significant differences between groups in MSA (≥80 years-old: 9.70 mm^2^ [7.63–12.20] vs. <80 years-old: 9.52 mm^2^ [7.30–11.40], p = 0.92). SE was also comparable between groups (≥80 years-old: 73% [67–78] vs. <80 years-old: 73% [62–81], p = 0.55). The AI at the MSA as well as the presence of persistent calcium fractures were also similar between groups (p = 0.16 and p = 0.52, respectively).

**Table 3:** Intracoronary imaging characteristics.

	≥80 years (n = 70)	<80 years (n = 224)	p-value^a^
**Intracoronary imaging used**	70/147 (47.6)	236/436 (54.1)	0.47
**Intracoronary imaging devices**	70	236	
IVUS	69 (98.6)	211 (89.4)	**<0.01**
OCT	1 (1.4)	25 (11.6)	**0.01**
**Reference vessel diameter** (mm)	4.1 ± 0.1	4.1 ± 0.1	0.91
**Pre-IVL minimum lumen diameter** (mm)	1.9 [1.7–2.4]	1.9 [1.7–2.2]	0.78
**Pre-IVL Area stenosis** (%)	68 [60–78]	72 [60–79]	0.19
**Max persistent Ca^2+^ angle** (^o^)	360 [220–360]	360 [270–360]	0.40
**Post minimum stent area** (mm^2^)	9.70 [7.63–12.20]	9.52 [7.30–11.40]	0.92
**Post stent expansion at MSA** (%)	73 [67–78]	74 [62–81]	0.55
**Post asimmetricy index at MSA**	0.15 [0.10–0.24]	0.15 [0.07–0.21]	0.16
**Persistent Ca^2+^ fracture**	34 (46.5)	109 (48.7)	0.52

Values are mean ± SD, median (IQR) or n (%).

IVUS, intravascular ultrasound; MSA, minimum stent area; OCT, optical coherence tomography.

Bold values indicate statistical significant.

a p-values were calculated to compare outcomes between patients aged ≥80 years and those aged <80 years.

### Procedural and clinical outcomes

**Fig. 1:**
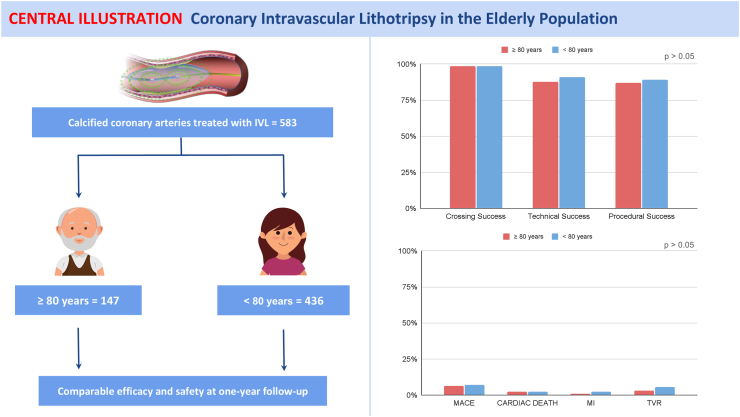
Intravascular lithotripsy showed comparable technical outcomes and one-year adverse events between advanced age (≥80 years old) and younger (<80 year old) population.

Table 4 shows procedural and clinical outcomes. Technical success rates were similar between the ≥80 years-old and <80 years-old groups, reaching 87.8% in the ≥80 years-old group and 90.6% in the <80 years-old group (p = 0.32). Similarly, procedural success rates were not significantly different, being 87.1% in the ≥80 years-old group and 89.0% in the <80 years-old group (p = 0.85). The incidence of MACE during hospitalis ation was low, occurring in 1.4% of the patients in the ≥80 years-old group and 2.1% in <80 years-old group (p = 0.59). Rates of cardiac death were also similar for both (1.4% vs. 1.6%, p = 0846). At 30-days, the rates of MACE were still low, with 2.0% in the ≥80 years-old group and 3.2% in the <80 years-old group (p = 0.47). Cardiac death and MI were infrequent, occurring in 1.4% and 0% of ≥80 years-old patients, compared to 1.6% and 1.1% in the <80 years-old group (p = 0.19 and p = 0.40, respectively). At 1-year follow-up, MACE rates were still comparable between both groups (6.1% vs. 8.0%, p = 0.51) (Fig. 1). Cardiac death occurred in 2.8% of ≥80 years-old patients and 2.1% of <80 years-old patients (p = 0.64). TVR rates were 2.7% in the ≥80 years-old group and 5.5% in the <80 years-old group (p = 0.36).

**Table 4:** Technical and clinical outcomes.

	≥80 years (n = 147)	<80 years (n = 436)	RR (95% CI)^a^	p-value^b^
**Technical success**	129 (87.8)	395 (90.6)	0.97 (0.91–1.04)	0.32
**Procedural success**	128 (87.1)	388 (89.0)	0.98 (0.91–1.05)	0.85
**In-hospital**				
MACE	2 (1.4)	9 (2.1)	0.66 (0.14–3.02)	0.59
Cardiac death	2 (1.4)	7 (1.6)	–	0.84
MI	0 (0)	0 (0)	–	–
TVR	0 (0)	2 (0.5)	–	0.41
**30-days**				
MACE	3 (2.0)	14 (3.2)	0.64 (0.19–2.18)	0.47
Cardiac death	2 (1.4)	7 (1.6)	–	0.84
MI	0 (0)	5 (1.1)	–	0.19
TVR	1 (0.7)	7 (1.6)	–	0.40
**6-months**				
MACE	6 (4.1)	27 (6.2)	0.66 (0.28–1.56)	0.45
Cardiac death	4 (2.7)	8 (1.8)	–	0.64
MI	1 (0.7)	8 (1.8)	–	0.32
TVR	2 (1.4)	18 (4.1)	–	0.11
**12-months**				
MACE	9 (6.1)	35 (8.0)	0.76 (0.38–1.55)	0.51
Cardiac death	4 (2.7)	9 (2.1)	–	0.64
MI	2 (1.4)	10 (2.3)	–	0.49
TVR	4 (2.7)	24 (5.5)	–	0.17

Values are reported as n (%). p-values were calculated to compare outcomes between patients aged ≥80 years and those aged <80 years.

MACE, major adverse cardiac events; MI, myocardial infarction; TVR, target vessel revascularization.

a RR and CI for ≥80 vs. < 80 years.

b The p-value were calculated to compare outcomes between the two considered populations.

## Discussion

**Fig. 2:**
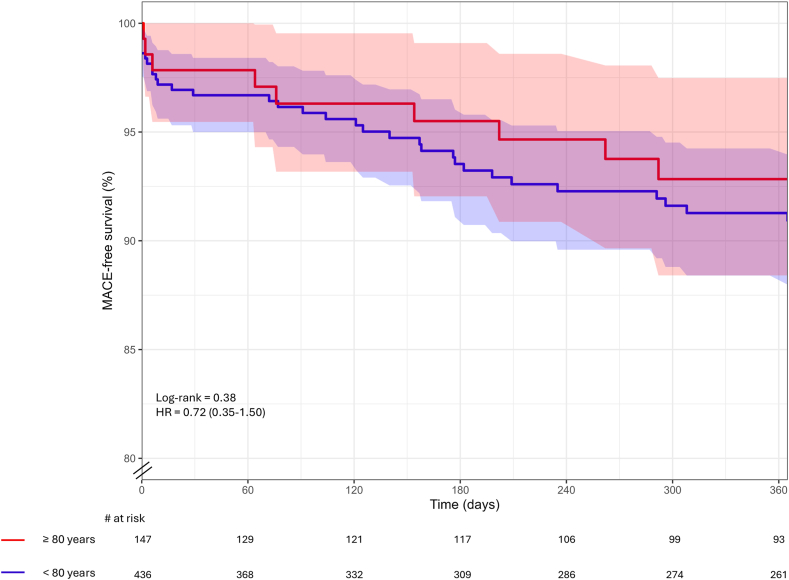
Kaplan–Meier curves showing 1-year major adverse cardiac event (MACE) outcomes in patients aged ≥80 years vs. <80 years undergoing intravascular lithotripsy (IVL). The curves demonstrate no significant difference in event-free survival between the two age groups.

The main purpose of this study was to evaluate the role of IVL in the treatment of calcific coronary lesions in the patients of advanced age, specifically in patients over 80 years of age. The key findings found in the study include: 1) technical and procedural success were similar in the ≥80 years-old and <80 years-old patient groups; 2) in-hospital and 1 year follow-up MACE were comparable between the two considered groups. 3) The rate of procedural complications was extremely low and comparable between the elderly and younger patients (Fig. 2). Results were consistent in the sensitivity analysis (Tables S1–S6).

With increasing life expectancy, the number of patients of advanced age requiring PCI is rising.^1^ However, performing PCI in this specific population presents additional challenges, like increased comorbidities and coronary anatomy, placing them at a higher risk of complications.^3^, ^4^, ^5^ Nevertheless, despite one-third of all PCIs being performed in patients over 75 years of age, patients of advanced age are frequently underrepresented or excluded from major clinical trials, leading to limited evidence and reliance on data extrapolated from younger populations.^1^ In a patient-pooled analysis of the Disrupt CAD studies, the mean age was 71.8 years.^17^ Thus, there is a gap in the evidence to support the use of IVL therapy in the advanced age.

Aging is strongly associated with an increased prevalence and severity of CAC, primarily due to degenerative processes and a high burden of comorbidities.^18^, ^19^, ^20^ The severity of CAC is a well-established predictor of stent underexpansion, a phenomenon that has been directly linked to stent thrombosis, target vessel failure and long-term MACE.^8^^,^^9^^,^^21^ Consequently, adequate lesion preparation is paramount to ensure optimal stent expansion and apposition.

In balloon-uncrossable lesions, atherectomy remains the primary modality for lesion modification. However, existing literature indicates that the use of rotational atherectomy (RA) in patients aged ≥80 years-old is associated with an increased risk of 30-day mortality.^1^ While this observation necessitates further validation through randomised controlled trials to control for potential confounders, it is likely that the increased mortality risk is partially attributable to the greater comorbidity burden in elderly individuals.

For balloon-crossable lesions, IVL has emerged as a safe and effective alternative.^10^^,^^22^ Previous research has demonstrated that IVL significantly improves coronary compliance, which in turn facilitates stent expansion.^11^^,^^12^ The findings of the present study confirm that IVL maintains its efficacy and safety profile in patients aged ≥80 years-old, thereby extending its applicability to this vulnerable population. This observation is particularly significant given that, within our registry, patients of advanced age exhibited worse baseline clinical characteristics, including lower LV-EF and reduced GFR. Additionally, higher SYNTAX scores among the advanced age cohort indicate an increased complexity of coronary artery disease (CAD), coupled with a higher frequency of IVL use in left main (LM) lesions. These factors contribute to a greater predisposition to periprocedural complications, including contrast-induced nephropathy and bleeding complications related to antithrombotic therapy. Our data suggest a preference for IVUS over OCT in patients of advanced age, a strategy that aligns with the need to minimize contrast exposure in this high-risk population.

Despite these inherent risks, our findings indicate no significant differences in intraprocedural complication rates between younger and patients of advanced age. Specifically, the incidence of major intraprocedural events, including D-F type dissections, vessel perforation, and abrupt vessel closure, remained low, with most complications occurring independently of IVL use. Furthermore, both in-hospital and long-term MACE rates were comparable between age groups.

Notably, we observed a numerically higher rate of TVR in the younger cohort. While this finding requires further investigation, it is plausible that clinicians exercised greater caution in managing patients of advanced age, favoring a more conservative approach over repeat revasculasisation, which may have contributed to an underestimation of adverse events in the older population.

Taken together, these findings underscore the safety and efficacy of IVL in elderly patients with heavily calcified coronary lesions. Despite their greater comorbidity burden and procedural complexity, IVL does not appear to confer additional procedural risk. Therefore, IVL might be considered as a preferred strategy for the management of calcified, balloon-crossable lesions in the advanced age, particularly in light of its favorable safety profile and the critical need to minimize procedural complications in this fragile population.

The present study has limitations. First, although a direct comparison between IVL and other calcium modification devices among the population of advanced age was beyond the scope of our study, such an analysis would be valuable in better contextualizing IVL's relative efficacy and safety in the percutaneous treatment of coronary calcified lesions. Second, despite the recognised importance of intracoronary imaging in guiding complex PCI, its use in our study was suboptimal, which may have influenced lesion assessment and optimization of stent deployment. Third, loss to follow-up and the relatively unpowered sample size for clinical outcomes reduces the robustness of our conclusions and their generalizability to broader clinical practice.

In conclusion, IVL demonstrates an excellent safety and efficacy profile for the treatment of calcified coronary lesions in patients with advanced age. It achieves high technical success while maintaining a low incidence of device-related adverse events, reinforcing its role as a viable and preferred strategy in this high-risk population.

## Contributors

FO and PVG contributed equally to this work. They were involved in the literature search, conceptualisation, data curation, formal analysis, and both original drafting and editing of the manuscript. MJHVO and AP contributed to data curation, methodology development, software implementation, and manuscript editing. BB provided supervision and was responsible for visualisation, including the preparation of figures and tables. VP and GM contributed to data curation and provided study resources. BEC, ADL, JK, HG, TV, and AMM contributed study resources and assisted with visualisation. FVDK, JWJ, and IAA contributed to supervision, editing, validation, and visualisation. JMMC was responsible for conceptualisation, funding acquisition, project administration, provision of resources, supervision, and manuscript editing. Both JMMC and JWJ directly accessed and verified the underlying data reported in the manuscript. All authors agreed to submit the manuscript for publication.

## Data sharing statement

Data is available upon reasonable request to the corresponding author.

## Declaration of interests

The Department of Cardiology of the Leiden University Medical Center received unrestricted research grants from Abbott Vascular, Bayer, Biotronik, Boston Scientific, Edwards Lifesciences, GE Healthcare and Medtronic. B.E.P.M. V. Paradies received unrestricted research grants from Abbott Vascular, SMT and Terumo via the Institution and consultancy fee from Abbott Vascular, Novo Nordisk, SMT, Elixir Medical, Boston Scientific. B. Claessen received consultancy fees from Abiomed, Abbott Vascular, Amgen, BBraun, Boston Scientific, Philips and Sanofi and received research funding from Philips, Novo Nordisk, BBraun and Infraredx. T. Vossenberg received consulting fees from Boston Scientific and speaker fees from Edwards lifescience and Medtronic. F. van der Kley received consultancy fees from Edwards Lifesciences and Abbott Vascular. JW Jukema/his department has received research grants from and/or was speaker (with or without lecture fees) on a.o. (CME accredited) meetings sponsored/supported by Abbott, Amarin, Amgen, Athera, Biotronik, Boston Scientific, Dalcor, Daiichi Sankyo, Edwards Lifesciences, GE Healthcare Johnson and Johnson, Lilly, Medtronic, Merck-Schering-Plough, Novartis, Novo Nordisk, Pfizer, Roche, Sanofi Aventis, Shockwave Medical, the Netherlands Heart Foundation, CardioVascular Research the Netherlands (CVON), the Netherlands Heart Institute and the European Community Framework KP7 Programme. I.Al Amri received Speaker fees from Penumbra Inc and Medtronic. JM. Montero received a research grant from Shockwave Medical and speaker fees from Abiomed, Boston Scientific and Penumbra Inc. The remaining authors have no conflicts of interest to declare.
