# Supplementary material for: Intravascular lithotripsy for the treatment of calcified coronary lesions in individuals of advanced age: a post-hoc analysis of the multicentre, prospective BENELUX-IVL study
Source: eClinicalMedicine. 2025 Jul 4;85:103342. doi: 10.1016/j.eclinm.2025.103342 (PMC12271790; doi:10.1016/j.eclinm.2025.103342)
Supplement: Supplement Materials [file mmc1.docx]

| **Table S1 Baseline characteristics** | | | | |
| --- | --- | --- | --- | --- |
|  | **≤. 65 years**  (n=116) | **65-80 years** (n=320) | **≥ 80 years** (n=147) | **p-value‡** |
| **Age**, yrs | 60 [56-63] | 73 [70-76] | 83 [81-85] | **< 0.01** |
| **Female** | 28 (24.1) | 81 (25.3) | 43 (29.3) | 0.58 |
| **BMI** | 26.6 [23.5-30.7] | 26.9 [24.2-29.4] | 25.6 [23.7-28.0] | **0.03** |
| **Hypertension** | 75 (64.7) | 223 (69.7) | 116 (78.9) | **0.03** |
| **Dyslipidemia** | 70 (60.3) | 178 (55.6) | 68 (46.3) | 0.07 |
| **Smoking history** | 57 (49.1) | 139 (43.4) | 46 (31.2) | **0.01** |
| **Diabetes Mellitus** | 32 (27.6) | 118 (36.9) | 43 (29.3) | 0.11 |
| **LV-EF** (%) | 55 [38-55] | 55 [45-55] | 52 [40-60] | 0.14 |
| **Syntax score** | 15 [10-24] | 20 [12-29] | 23 [15-32] | **< 0.01** |
| **Chronic Kidney Disease**  **(**eGFR <60 ml/min/1.73 m^2^**)** | 16 (13.8) | 95 (29.7) | 65 (44.2) | **< 0.01** |
| **GFR** (ml/min) | 89 [73-90] | 70 [55-85] | 61 [46-74] | **< 0.01** |
| **Previous PCI** | 60 (51.7) | 149 (46.6) | 56 (38.0) | 0.07 |
| **Previous CABG** | 21 (18.1) | 65 (20.3) | 19 (12.9) | 0.15 |
| **Previous MI** | 51 (44.0) | 120 (37.5) | 41 (27.9) | **0.03** |
| **Previous Stroke/TIA** | 9 (7.8) | 38 (11.9) | 28 (19.0) | **0.02** |
| **Clinical presentation**  Stable angina  Unstable angina  NSTEMI  STEMI  Other | 57 (49.1)  10 (8.6)  25 (21.6)  11 (9.5)  13 (11.2) | 164 (51.3)  34 (10.6)  67 (20.9)  25 (7.8)  30 (3.1) | 60 (40.8)  19 (12.9)  49 (33.3)  7 (4.8)  12 (8.2) | 0.11 |
| **Angina pectoris ꝉ**  No angina  Class I  Class II  Class III  Class IV  Unknown | 16 (13.8)  3 (2.6)  42 (36.2)  30 (25.9)  7 (6.0)  18 (15.5) | 36 (11.3)  13 (40.7)  121 (37.8)  70 (21.9)  31 (9.7)  47 (14.7) | 22 (15.0)  5 (3.4)  38 (25.9)  40 (27.2)  22 (15.0)  19 (12.9) | 0.22 |
| **Anti-ischemic medication**  Beta-Blockers  Nitrates | 65 (56.0)  36 (31.0) | 199 (62.2)  88 (27.5) | 86 (58.5)  42 (28.6) | 0.46  0.77 |

*Values are mean ± SD , median (IQR) or n (%). ꝉ according to the Canadian Cardiovascular Society grading of angina pectoris. ‡ p-values were calculated for the three considered populations. eGFR = estimated glomerular filtration rate (using the MDRD [Modification of Diet in Renal Disease] formula); DOAC = direct oral anticoagulants; eGFR = estimated glomerular filtration rate); NSTEMI = non ST-elevation myocardial infarction; PCI=percutaneous coronary intervention; STEMI = ST-elevation myocardial infarction;*

| **Table S2 Procedural characteristics** | | | | |
| --- | --- | --- | --- | --- |
|  | **≤. 65 years**  (n=116) | **65-80 years** (n=320) | **≥ 80 years** (n=147) | **p-value‡** |
| **Procedural time** (min) | 81 [62-106] | 81 [59-111] | 80 [58-111] | 0.95 |
| **Contrast volume** (ml) | 177 [130-220] | 161 [131-239] | 180 [120-238] | 0.82 |
| **Inotropes** | 3 (2.6) | 8 (2.5) | 8 (5.4) | 0.21 |
| **Vasopressor** | 3 (2.6) | 7 (2.2) | 5 (5.4) | 0.73 |
| **Need for Mechanical support**  IABP  Impella  VA-ECMO | 3 (2.6)  2 (1.7)  1 (0.9)  1 (0.9) | 8 (2.5)  0 (0)  5 (1.6)  3 (0.9) | 5 (5.4)  0 (0)  5 (5.4)  0 (0) | 0.84 |
| **Access**  radial  femoral | 90 (77.6)  29 (25.0) | 251 (78.4)  76 (23.8) | 107 (72.8)  40 (27.2) | 0.59  0.63 |
| **Target lesion**  left main  left anterior descending artery  left circumflex  right coronary artery  venous graft | 117  9 (7.8)  45 (38.5)  20 (17.1)  50 (42.7)  1 (0.9) | 342  34 (10.6)  118 (34.5)  58 (17.0)  121 (35.3)  3 (0.9) | 153  24 (15.7)  64 (41.8)  22 (13.8)  41 (26.8)  2 (1.3) | 0.08  0.49  0.80  **0.02**  0.89 |
| **Bifurcation** | 24 | 70 | 41 | 0.26 |
| **CTO** | 16 | 25 | 4 | **< 0.01** |
| **Ostial lesions** | 21 | 84 | 43 | 0.15 |
| **In-stent** | 47 | 110 | 28 | **< 0.01** |
| **Rotational atherectomy** (before IVL) | 13 | 45 | 17 | 0.75 |
| **Cutting Balloon** (before IVL) | 0 | 5 | 0 | 0.14 |
| **Pre-IVL high-pressure dilatation** | 109/117 (93.1) | 313/342 (91.5) | 140/153 (91.5) | 0.87 |
| **Pre-IVL largest balloon** (mm) | 3.0 [3.0-3.5] | 3.0 [3.0-3.5] | 3.0 [2.5-3.5] | 0.12 |
| **Pre-IVL maximum pressure dilatation** (atm) | 20 [18-22] | 20 [18-22] | 18 [16-20] | 0.45 |
| **IVL crossing success** | 109 (94.0) | 308 (96.3) | 140 (95.2) | 0.77 |
| **IVL pulses delivered**  < 80  80  > 80 | 90  59  4 | 152  156  14 | 78  70  5 | 0.23 |
| **Maximum diameter IVL balloon** (mm) | 3.5 [3.5-4.0] | 3.5 [3.0-3.5] | 3.5 [3.0-4.0] | 0.09 |
| **Post-IVL high-pressure dilatation** | 109/117 (93.1) | 314/342 (91.8) | 140/153 (91.5) | 0.85 |
| **Post-IVL largest balloon** (mm) | 3.5 [3.5-4.0] | 3.5 [3.5-4.0] | 3.5 [3.0-4.0] | 0.22 |
| **Post-IVL maximum pressure dilatation** (atm) | 20 [18-22] | 20 [16-22] | 18 [16-20] | 0.28 |
| **Drug eluting balloon** | 14 | 23 | 9 | 0.12 |
| **Total stent length** (mm) | 38 [24-60] | 42 [26-60] | 34 [24-49] | **0.02** |
| **Stent maximum diameter** (mm) | 3.5 [3.5-4.0] | 3.5 [3.5-4.0] | 3.5 [3.0-4.0] | 0.87 |
| **Intraprocedural complications**  severe dissections (D - E - F)  abrupt vessel closure  perforation  need for cover stent  tamponade  Hemodynamic instability  **Complication IVL-related** | 4 (3.4)  0 (0)  0 (0)  3 (2.6)  3 (2.6)  1 (0.9)  1 (0.9)  0 (0) | 21  7  3  3  2  0 (0)  4  3 | 10  3  2  2  1  0 (0)  4  4 | 0.43  0.14 |

*Values are mean ± SD or median (IQR). ‡ p-values were calculated for the three considered populations. IVL = intravascular lithotripsy.*

| **Table 3 Intracoronary imaging characteristics** | | | | |
| --- | --- | --- | --- | --- |
|  | **≤. 65 years**  (n= 65) | **65-80 years** (n= 171) | **≥ 80 years**  (n= 70) | **p-value‡** |
| **Intracoronary Imaging used** | 65/116 (57.8) | 171/320 (53.4) | 70/147 (47.6) | 0.35 |
| **Intracoronary imaging devices**  IVUS  OCT | 65  56 (86.2)  9 (13.8) | 171  145 (90.6)  16 (9.4) | 70  69 (98.6)  1 (1.4) | **< 0.01**  **< 0.01** |
| **Reference vessel diameter** (mm) | 4.1 ± 0.1 | 4.0 ± 0.1 | 4.1 ± 0.1 | 0.48 |
| **Pre-IVL Minimum lumen diameter** (mm) | 1.9 [1.6-2.3] | 1.9 [1.7-2.3] | 1.9 [1.7-2.4] | 0.94 |
| **Pre-IVL Area stenosis** (%) | 76 [62-81] | 71 [61-79] | 68 [60-78] | 0.18 |
| **Max persistent Ca^2+^ angle** (^o^) | 360 [266-360] | 360 [270-360] | 360 [220-360] | 0.67 |
| **Post Minimum stent area** (mm^2^) | 9.93 [8.35-12.30] | 9.40 [7.38-11.20] | 9.70 [7.63-12.20] | 0.29 |
| **Post Stent expansion at MSA** (%) | 76 [62-84] | 73 [68-79] | 73 [67-78] | 0.97 |
| **Post Asimmetricy index at MSA** | 0.15 [0.09-0.22] | 0.14 [0.06-0.21] | 0.15 [0.10-0.24] | 0.33 |
| **Persistent Ca^2+^ fracture** | 31 (47.7) | 78 (45.6] | 34 (46.5) | 0.90 |

*Values are mean ± SD , median (IQR) or n (%). ‡ p-values were calculated for elderly vs the non-elderly population.* *MSA = minimum stent area.*

| **Table S4 Technical and clinical outcomes** | | | | |
| --- | --- | --- | --- | --- |
|  | **≤. 65 years**  (n=116) | **65-80 years** (n=320) | **≥ 80 years** (n=147) | **p-value‡** |
| **Technical success** | 102 (87.9) | 293 (91.6) | 129 (87.8) | 0.33 |
| **Procedural success** | 101 (87.1) | 287 (89.7) | 128 (87.1) | 0.73 |
| **In-hospital:**  MACE  Cardiac death  MI  TVR | 1 (0.9)  1 (0.9)  0 (0)  0 (0) | 8 (2.5)  6 (1.9)  0 (0)  2 (0.6) | 2 (1.4)  2 (1.4)  0 (0)  0 (0) | 0.46  0.73  -  0.44 |
| **30-days:**  MACE  Cardiac death  MI  TVR | 3 (2.6)  1 (0.9)  2 (1.8)  2 (1.9) | 11 (3.4)  6 (1.9)  3 (0.9)  5 (1.6) | 3 (2.0)  2 (1.4)  0 (0)  1 (0.7) | 0.69  0.73  0.31  0.70 |
| **6-months:**  MACE  Cardiac Death  MI  TVR | 7 (6.0)  1 (0.9)  3 (2.6)  6 (5.2) | 20 (6.3)  7 (2.2)  5 (1.6)  12 (3.8) | 6 (4.1)  4 (2.7)  1 (0.7)  2 (1.4) | 0.63  0.56  0.46  0.22 |
| **12-months:**  MACE  Cardiac Death  MI  TVR | 12 (10.3)  1 (0.9)  4 (3.4)  11 (9.5) | 23 (7.2)  8 (2.5)  6 (1.9)  13 (4.1) | 9 (6.1)  4 (2.7)  2 (1.4)  4 (2.7) | 0.41  0.53  0.47  **0.03** |

*Values are reported as n (%). ‡ p-values were calculated for the elderly vs the non-elderly population. MACE: major adverse cardiac events; TVR: target vessel revascularization;*

| **Table S5 QCA angiographic characteristics** | | | |
| --- | --- | --- | --- |
|  | **≥ 80 years** (n= 126) | **< 80 years** (n= 381) | **p-value‡** |
| **Reference vessel diameter** (mm) | 3.2 [2.70-3.60] | 3.2 [2.80-3.70] | 0.62 |
| **Minimum lumen diameter** (mm) | 1.0 [0.6-1.3] | 0.9 [0.5-1.4] | 0.20 |
| **Pre PCI Diameter stenosis** (%) | 68 ± 1.6 | 68 ± 1.1 | 0.91 |
| **Post PCI Minimum lumen area** (mm^2^) | 6.0 [4.2-8.6] | 6.3 [4.9-8.4] | 0.25 |
| **Acute gain** (mm) | 1.8 [1.30-2.30] | 1.8 [1.30-2.40] | 0.46 |

*Values are mean ± SD or median (IQR).*

| **Table S6 Univariate Logistic regression** | | | |
| --- | --- | --- | --- |
| **Indipendet variable** | **Dependent variable** | **OR (95% CI)** | **p-value** |
| *Age* | *Technical success* | *0.99 ( 0.96–1.02)* | *0.44* |
| *Age* | *Procedural success* | *0.99 (0.97–1.02)* | *0.60* |

*Results of univariate logistic regression analysis evaluating the association between age and technical or procedural success. Odds ratios (OR) are reported with corresponding 95% confidence intervals (CI) and p-values.*
